# Supplementary material for: Two-years antibody responses following SARS-CoV-2 infection in humans: A study protocol
Source: PLoS One. 2022 Aug 16;17(8):e0272690. doi: 10.1371/journal.pone.0272690 (PMC9380924; doi:10.1371/journal.pone.0272690)
Supplement: Attachment — Submitted filename: Response to Reviewers-round2.docx [file pone.0272690.s002.docx]

Dear Academic Editor
PLOS ONE

Thank you for giving us the opportunity to submit a second round revised draft of our manuscript titled “Two years antibody responses following SARS-CoV-2 infection in humans: a study protocol” to *PLOS ONE*.

Again, we appreciate the time and effort that you and the reviewers have dedicated to providing your valuable feedback after our first response. We have highlighted the changes within the manuscript and you can identify the changes from track changes. Here is a point-by-point response to the reviewers’ comments and concerns.

**Reviewer #1:** *Reviewer #1: Thank you for revising the manuscript according to my comments. The revision has made significant improvements to the overall quality of the manuscript, especially in the Methodology section. I think the manuscript can now be accepted for publication.*

Response: Thank you so much for your positive comment.

**Reviewer #2:** *Although the article has scientific rigor, several minor flows need to be corrected before publication.*

*Minor comments:*

**Comment 1:**

*Introduction: The introduction section seems missing important information. This section needs profound modification with more Up-to-date references.*

Response: Thank you for your valuable feedback. We revised the introduction section (highlighted)

**Comment 2:**

*Discussion: The discussion section can improve.*

Response: Thank you so much. We appreciate your comment. We revised the discussion section (highlighted)

We look forward to hearing from you in due time regarding our re-submission.

Sincerely,

Lutfan Lazuardi, MD, PhD

Corresponding author

On behalf of all authors

Juli 16, 2022
